# Parental experiences of raising an autistic child in Ireland: A qualitative thematic analysis

**DOI:** 10.1177/13623613241277040

**Published:** 2024-09-12

**Authors:** Sally Whelan, Niall Caulfield, Sinéad O’Doherty, Arlene Mannion, Geraldine Leader

**Affiliations:** University of Galway, Ireland

**Keywords:** autism, autism spectrum disorder, coping strategies, parental challenges, parental stress, qualitative methodology, support services

## Abstract

The quality of life of autistic children and their parents is impacted by the stress they experience, their coping strategies and the availability of professional health, social and educational support services. Recent changes in the structural organisation of child disability professional supports in Ireland mean that in-depth knowledge about current experiences of parenting autistic children is necessary. This qualitative study explored parents’ perceptions and experiences regarding their challenges, stress levels, coping strategies and professional support services. Semi-structured in-depth interviews were conducted with six parents of autistic children aged 4 to 16 years. Thematic analysis identified three core themes: ‘The Autism Journey: Challenges and Rewards’, ‘Navigating a Flawed Support System’ and ‘The Importance of Social and Professional Supports’. Findings emphasised that parents face endless challenges in caring for autistic children. Dealing with autism-based support services, however, is the greatest stressor experienced by parents. It revealed that the system to access services is experienced as difficult and parents consider it is operating inadequately. This reveals a pressing need to improve systems that provide professional support services to autistic children and their families.

**Lay abstract:**

Parenting an autistic child can be a challenging experience. Parents of autistic children often require social and professional support to cope with the various stresses they encounter and to ensure their children achieve their optimal potential. Recently, the way professional supports are organised in Ireland has changed. Very little previous recent research has investigated parents’ experiences of raising an autistic child in Ireland. This study interviewed six parents asking them about their challenges, stress levels, coping strategies and their perceptions regarding professional support services. The data from these interviews were organised into themes. A major finding was that parents felt the healthcare system was failing to provide help for their children, and that support services in Ireland can cause more parental distress than dealing with their child’s difficulties. Other causes of parental stress included the child’s behaviours that they found challenging, stigma, a lack of awareness about autism and isolation. This study shows that both autistic children and their parents are at increased risk of developing mental health problems due to a flawed healthcare system that requires improvement urgently.

## Introduction

Autism is a neurodevelopmental condition that impacts a person’s cognitive, language, sensory perceptions and social abilities (Lee et al., 2017). Worldwide, approximately 1 in 59 children is autistic (Baio et al., 2018). How autism presents for individuals, and its severity, is highly variable. Emotional and behavioural difficulties are common and autistic children and adolescents display anxiety, aggression and depressive symptoms (Hastings et al., 2021; Lindor et al., 2019). In addition, autistic individuals frequently experience co-occurring mental and physical health conditions, including attention-deficit/hyperactivity disorder (ADHD), obsessive-compulsive disorder (OCD), oppositional defiance disorder (ODD), epilepsy, obesity and diabetes (Joshi et al., 2010; Malik-Soni et al., 2022; Mosner et al., 2019).

Autistic children vary regarding the level of support that they require (Malik-Soni et al., 2022), but many require professional support services to ensure they thrive and reach their optimal potential (Blanc et al., 2021; Ilias et al., 2018). However, finding knowledgeable health, social and educational professionals who can assess autism and understand the unique challenges that accompany raising an autistic child can prove difficult (Brown et al., 2010; Hus & Segal, 2021). Indeed, physicians can find it difficult to diagnose autism due to the heterogeneity of the condition (Hus & Segal, 2021; Weitlauf et al., 2014). It is recommended that autism is diagnosed through assessments carried out by a multidisciplinary team (Rabbitte et al., 2017). The combination of waiting to receive an assessment and worries about the scarcity of autism-specific interventions often elicits anxiety in parents (Connolly & Gersch, 2013; Höfer et al., 2019).

For some parents, raising an autistic child can be an all-encompassing experience (Cooke et al., 2020) and the impact of autism on parents can be complex, far-reaching and enduring. Up to 5% of autistic people may require some level of assistance from caregivers for their lifetime, because they also have cognitive and/or adaptive limitations that hinder their capacity to live independently (Marsack-Topolewski, 2020; Volkmar & Pauls, 2003). Attempting to fulfil the needs of an autistic individual can place parents under immense pressure (Roquette Viana, 2021). Compared to parents of children with other developmental conditions, parents of autistic children experience greater levels of stress and lower levels of well-being (Ilias et al., 2018). Sources of parental stress are impacted by factors particular to the individual child. These include unpredictable and challenging behaviours (Ilias et al., 2018) such as self-injurious behaviour, hostility towards others and the destruction of property (Kanne & Mazurek, 2011; Lecavalier, 2006; Sullivan et al., 2019). Stress is also impacted by the extent of the child’s autism, whether the child has sleep problems (Mannion & Leader, 2023). Factors external to the child also impact parental stress. The societal stigma that surrounds autism impacts parents through the criticism and judgement they receive regarding their child’s behaviour in public settings (Bonis, 2016; Lutz et al., 2012; Neely-Barnes et al., 2011; Swaab et al., 2021). Increased parental stress is also associated with the paucity of professional and social support available (Al-Oran et al., 2022).

Unfortunately, support from friends and family members can be elusive, with many parents reporting strained relationships with other people due to having an autistic child (Lodder et al., 2019). Professional support, however, can benefit parents in several ways. These supports can help parents accept the nature of autism through the provision of skills that enable them to react more appropriately to their child’s behaviour. In addition, professional support can teach parents to self-advocate to ensure they have access to the services required to adequately support their child (Banach et al., 2010; Leadbitter et al., 2021). Social support and mental health services can also help parents of autistic children to manage their stress (Ilias et al., 2018). Particularly, parents can be supported to develop healthy coping strategies to manage the daily challenges they experience. Lazarus and Folkman (1984), defined coping as a process by which behavioural and cognitive abilities are used to manage stressful situations (Al-Oran et al., 2022). They discuss two forms of coping strategies: problem-focused coping (e.g. engagement, positive reframing) and emotion-focused coping (e.g. avoidance, distraction and denial). Problem-focused coping is associated with more positive outcomes for parents of autistic children (Higgins et al., 2022). Problem-based coping alongside social support–seeking strategies has been deemed effective at reducing stress and increasing parents’ quality of life (Vernhet et al., 2018).

In the Republic of Ireland, autism occurs in approximately 1% of the population (Roddy & O’Neill, 2020). In Ireland, state-funded support services for autistic children and their families are provided by a publicly funded health and social care system organised by the Health Service Executive (HSE). To date, there has been relatively little research conducted to provide in-depth data to understand the experiences and perceptions of parents raising a child with autism in Ireland. Previous qualitative studies have focused on parental perspectives regarding diagnostic services (Langford et al., 2012; OBrien, 2021) and securing a diagnosis for daughters (Rabbitte et al., 2017). Byrne et al. (2018) described the experience of parenting Irish autistic children (n = 10) focusing on the relationships between family members and their interactions with the wider community. They found that parents emphasised experiencing blame and stigma, isolation and a sense of disconnection, and the need to be constantly vigilant and advocate for the child.

However, further investigation is currently needed because the context around raising a child in Ireland has changed in recent years. Prior to September 2021, the HSE provided services for children with disabilities based on their residential geographical area. In 2021, the HSE began reorganising service provision under the Progressing Disability Services (PDS) Programme. The PDS Programme aims to ensure timely, equitable access to quality services (HSE, 2023). Under the PDS children are allocated to a regional Children’s Disability Network Team (CDNT). Teams consist of social and healthcare professionals that include nurses, psychologists, speech and language therapists, occupational therapists, physiotherapists and medical personnel.

However, recent quantitative survey evidence suggests that the PDS roll-out has been problematic and waiting times to access services have increased (Inclusion Ireland, 2022). An examination of parental perceptions is currently needed, in this changed context, to promote the voices of parents and to provide an in-depth understanding of their current needs and their experiences parenting an autistic child in Ireland. This knowledge is necessary to inform policymakers and to guide clinical practice.

Therefore, this study aimed to explore, in-depth, the current perceptions and experiences of parenting an autistic child in Ireland. Its objectives were to explore how parents of autistic children cope in their everyday lives, to identify the challenges they face and to ascertain their perceptions regarding the level of service provision that is accessible to their children, and the impact that service provision has on their quality of life.

## Methodology

### Study design

A qualitative descriptive methodology was used because this approach can generate data to describe the subjective perspectives of parents concerning the ‘who, what, and where of events or experiences’ (Kim et al., 2017, p. 23).

### Community involvement statement

Community involvement occurred as the study was conceived following conversations between researchers (G.L., A.M. and S.W.) and parents of autistic children who are involved in autism advocacy groups. These discussions also guided the study design and the materials and techniques used during data collection. The latter were also piloted in two interviews with parents. The data collected during these interviews were not included in the final analysis, but the participant feedback was used to amend the final interview guides.

### Sample

In this study, the number of participants was determined using the principles of data saturation, as described further below. The participants were parents (n = 6) of autistic children (n = 8). Five mothers and one father participated who ranged in age from 43 to 62 years. Five out of six participants were married, and one was single. Two parents (participants 2 and 3) had two autistic children, while the remaining four parents had one autistic child. Table 1 presents the children’s demographic details and the corresponding parents. The children ranged in age from 4 to 16 years. Two of the eight children were nonverbal. They attended a range of educational settings. One child attended a school for children with special needs, two attended autism spectrum disorder (ASD) preschools and five attended mainstream primary school either with support workers or in dedicated ASD units.

**Table 1.** Children’s demographic details and corresponding parents.

Child’s gender *M/F*	Child’s age	School	Verbal/nonverbal (V/NV)	Parent participants (P)
F	16	Special School	NV	P 1
M	9	Mainstream ASD Unit	V	P2^a^
M	9	Mainstream ASD Unit	V	P2^a^
M	9	Mainstream with Support	V	P 3^a^
F	11	Mainstream with Support	V	P 3^a^
M	5	ASD Preschool	V	P 4
F	6	Mainstream with Support	V	P 5
M	4	ASD Preschool	NV	P 6

a Participants have two children with autism.

### Procedure

Ethical approval was granted by the Psychology Ethics Committee at University of Galway. Participants in this study had previously taken part in a quantitative study that explored parental satisfaction with service provision in Ireland (Cahill et al., n.d.). Upon completion of this previous study, participants had expressed interest in participating in a further qualitative in-depth interview about this topic. Participants in this previous study were initially recruited through advertisements placed in support groups on Facebook frequented by parents with autistic children.

At the start of the current study, participants were contacted via email. They were invited to read a study participant information sheet. The information sheet informed the potential participants about the study’s aims and methods, and it reminded them they could withdraw at any point. They were also invited to ask questions about the study. If they decided to participate parents were requested to complete a consent form and return this to the researcher. Then an interview time was arranged at the participant’s convenience.

### Data collection and data management

A total of six semi-structured interviews were carried out between May and June 2023. These lasted from 20 to 72 min. The interviews were conducted and recorded using Zoom virtual conferencing technology using the procedures described below.

The researcher sent a Zoom invitation via email to the participant shortly before the scheduled meeting. At the start of the interview the participant was greeted, rapport was established and then the researcher reminded the participant about the study’s aims, and their rights, as outlined by the information sheet. When the participants were ready to start, the researcher began recording. During the interviews, the researcher followed an interview guide. This was used to gently guide the conversation to relevant topics by asking open-ended questions (see Table 2). Having asked the guide questions the researcher followed up with prompts and relevant responses aiming to relax and encourage participants to elicit pertinent information. When the interview concluded, the researcher thanked the participants for their valuable contribution and said that they would receive a summary of the study results. Participants were invited to provide researchers with feedback on these results, if they wished to do so. In addition, participants were also offered a list of supportive/counselling professional services that they could access, should they wish to do so.

**Table 2.** Interview guide questions.

Number	Question
1	Can you start by telling me about your experiences of parenting [name of the child]?
2	Can you tell me about any particular things that may have been difficult or challenging for you while parenting [name of the child]?
3	Can you tell me about how you cope with any challenges or things that are difficult?
4	I would like to ask you about the supports and services you receive for [name of the child]?
5	How do you think your experience of the supports and services affects your stress levels and your quality of life?
6	Is there anything that we have not talked about which you think is important to mention?

Following the interview, the researcher uploaded the data recordings to OneDrive. These were saved in a password-encrypted file, on a password-protected computer. The researcher also made contemporaneous contextual notes about the interview.

After each interview, the researcher (N.C.) transcribed the recording. During which all identifiable data such as names were omitted, and pseudonyms were assigned in their place (e.g. participant 1). The accuracy of the transcriptions was ascertained through repeated listening to the recordings and checking the transcripts. Then, the transcripts were uploaded into the qualitative data management software NVivo 12 (Dhakal, 2022).

### Data analysis

Data analysis was conducted in NVivo 12 following specific guidelines of Clarke and Braun’s (2013) thematic approach to analysis. This allowed for patterns and themes in the data to be identified using six phases. First, the researcher (N.C.) familiarised themselves with the data, then initial codes were generated. After this, researchers (S.W. and N.C.) reviewed the data and the codes. At this stage researchers considered that data saturation had been achieved because there was repetition in the data and sufficient data for the researchers to ascertain the patterns in the data with confidence. Then NC gathered the codes into potential themes. After which, S.W. and N.C. again reviewed the analysis jointly to check that the themes encompassed all the data set, that they related accurately to each of the coded extracts and that no additional issues were identified that would warrant further data collection. Then both researchers defined and named the themes according to their characteristics. The final review of the analysis was undertaken by researchers (N.C., S.W. and S.O.) when the study report was written up.

### Research quality

To address reflexivity, before commencing this research the researchers (N.C. and S.W.) discussed at length their attitudes towards the topic. This discussion enabled them to identify their potential biases and how these may impact the research processes. After this, N.C. maintained a reflective diary throughout the research that was reviewed and discussed with S.W. This diary facilitated N.C. to reflect upon all the research processes. In addition, an audit trail was maintained using this diary and the NVivo software.

## Results

**Figure 1.**
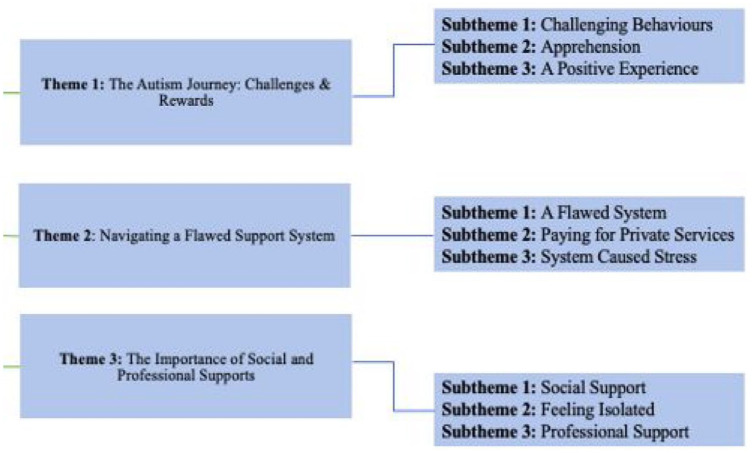
Thematic map displaying themes and subthemes.

Three core themes were identified: Theme 1: ‘The Autism Journey: Challenges and Rewards’, Theme 2: ‘Navigating a Flawed Support System’ and Theme 3: ‘The Importance of Social and Professional Supports’. These themes and subthemes are described below, and Figure 1 presents them in an illustrative summary.

### Theme 1

#### The autism journey: challenges and rewards

This theme describes the everyday challenges that parents experience raising an autistic child. It encompasses data relating to challenging behaviours and complex emotions such as the frustration and fear experienced in trying to meet children’s needs. It also includes parents’ rewarding experiences in raising an autistic child. It includes the following subthemes: Challenging Behaviours, Apprehension and A Positive Experience.

##### Subtheme 1: Challenging Behaviours

All participants shared their stories relating to the everyday struggles of parenting an autistic child. Each parent’s story was unique, emphasising that each child has their own specific needs. Reoccurring problem behaviours caused parents major distress. For example,

. . . she could have meltdowns. . . they’re daily. . . they’re exhausting for her, and they’re exhausting for me, that’s our life. (Participant 1)

Our greatest challenge is that the boys don’t get on. The boys are very physical, very aggressive. . . There are constantly sparks flying. . . (Participant 2)

When he gets out of the car, he has to touch the roof and he has to get out in his own time. You can’t rush him. It can be lashing rain and if he decides he doesn’t want to get out the car, he won’t get out the car. (Participant 4)

You get a lot of meltdowns and slapping, and he used to bite. . . a lot of frustration. . . but if he want something right away and you say no, not yet, then watch out! (Participant 6)

Some parents reported that the family must frequently adapt to accommodate the child’s needs to avoid meltdowns, which can be taxing.

Everything his way or not at all! He’s so used to getting his own way. . . We’d go out for walks. . . and if you didn’t go the way he wanted to go, then it’s full-on screaming, shouting, whatever. . . until he gets his own way sort of thing. (Participant 4)

##### Subtheme 2: Apprehension – Fear of the Unknown

Parents expressed great concern about the ‘uncertainty’ of their children’s behaviours. The potential for dangerous behaviours being exhibited by their children was a constant source of anxiety for some parents.

He’s got no real understanding of danger. . . you have to keep up with him when you’re out because there is a chance that out of the blue, he’ll just run onto the road. He’s done that a couple of times recently, giving me a heart attack! (Participant 4)

Parents are also apprehensive about what will happen to their children in the future, especially as they won’t be around to care for them forever.

I have no idea how (name of child) is going to be cared for when I move on, that’s not far away!. . . and of course, as you get older your ability to be energetic and provide care is less. . . so that’s a real problem as well. (Participant 1)

##### Subtheme 3: A Positive Experience – ‘There is a Positive Side’

Parents were eager to share their positive experiences. Some became emotional when describing how their child positively impacts their lives.

She is an absolute diamond. Yeah. She’s worth every bit of it. (Participant 1)

We adore the boys, and when we got their autism diagnosis, we went out for dinner! We went out and celebrated because. . . we saw it as a positive. . . we felt, okay, now we can get what the boys need. (Participant 2)

I wouldn’t change it for the world’. (Participant 3)

Parents conveyed that even though at times it can be a challenging and complex process, the experience can be extremely positive. Many parents found their children’s progress rewarding. For example,

This is where he’s at. And look at what he’s achieved. Look at how far he’s come. (Participant 4)

He is coming on, like the school he’s in is very, very good. So, we are noticing progress that way. (Participant 6)

### Theme 2

#### Navigating a ‘flawed’ support system

This theme describes the difficulties that parents experienced accessing health services and receiving desired treatments. It includes the following subthemes: A Flawed System, Paying for Private Services and System Caused Stress.

##### Subtheme 1: A Flawed System

Participants frequently expressed views that the system is flawed and performing inadequately. Their views about the system were almost entirely negative. Many parents highlighted the need for the system to be reformed:

It’s completely flawed, it’s beyond flawed. It needs to be completely torn down. . . I don’t think people realize how rotten the whole system is. . . (Participant 2)

One participant commented that the process of needs assessment has deteriorated since 2010, saying,

The process back then was so much better really, we’ve gone backward. . . absolutely gone backward, and it’s criminal. (Participant 1)

Some participants perceived that the system strategically employs barriers to support services because they simply cannot cope with the demand. For example, two participants said,

You feel that every time you’re making an application, the go-to will be, no, sorry, you don’t qualify. (Participant 1)

It’s the barriers in the schools and in the broader system. . . that are huge, huge obstacles. (Participant 3)

Participants also suggested that the system is inadequately responding to the needs of children because it is over capacity or inactive. Parents perceive their child as being passed between services:

They’re so overwhelmed with the numbers of assessments and needs; they don’t have the capacity to deliver proper disability services. (Participant 1)

Every service is passing them [the child] back and forth. . . We were bounced, and still are being bounced. . . It’s a paper trail, that’s all it is, nothing active going on. (Participant 2)

Participants particularly found it difficult to access information, immediately after their child’s diagnosis, regarding how to access early interventions and understand autism. They would have liked one place where they could access all the information that they needed. For example, participants said,

There isn’t one central place where you get that information. (Participant 2)

I would’ve preferred to get all the knowledge. . . but it just wasn’t there. . . Who can you contact on one information page? As opposed to getting information off all the parents. (Participant 5)

All I wanted was someone to hand me a book. . . have it all in one space. (Participant 6)

##### Subtheme 2: Paying for Private Services

Lack of access to support services frequently left parents with no alternative other than to pay for private services. This caused some parents financial strain:

. . . we had to go down the private route because we weren’t going to wait four years, and that’s very costly. (Participant 3)

. . . the public system doesn’t have the capacity to do [a cognitive assessment]. . . . So, I have to go private for that, which is another 800 euro. (Participant 6)

One participant was shocked to be advised by a healthcare worker to spend their monthly Domiciliary Care Allowance (DCA) towards paying for private services.

. . . they actually said, we actually recommend parents to spend their DCA money, going private. . . (Participant 4)

##### Subtheme 3: System Caused Stress

Navigating the system was time-consuming and it caused all the participants hardship and stress.

It’s a harrowing experience. . . it would bring a tear to a glass eye. . . (Participant 1)

Yeah, my experience, it has been quite overwhelming. (Participant 3)

Many participants expressed their frustrations with the amount of paperwork involved when trying to access services which elevated their levels of stress. For example, participants said,

. . . they constantly send you out more paperwork to fill out. . . it’s been like 90% paperwork and 10% service. (Participant 3)

Parents with two autistic children (participants 2 and 3) and one autistic child (participant 1) stated that dealing with the HSE system has been a greater stressor for their family than the condition itself:

. . . dealing with the professionals has been the most challenging thing. It hasn’t been autism itself. (Participant 2)

Not that my kids are overwhelming,. . . It’s been the system that’s been the hardest part, the system, and the school. (Participant 3)

It’s actually more difficult to get the support than it is to look after the child. (Participant 1)

Some parents reported that advocating for their child to receive the necessary support is an extremely draining experience and one that elicits feelings of anger, frustration and anxiety.

You’ve got to keep hammering away. . . to advocate for your child, and sometimes that is the most difficult part of being a parent of a person with special needs. (Participant 1)

The system should not be dependent on parents shouting the loudest. . . I shout very loud and we’re still not getting anywhere. . . it’s very disheartening. (Participant 2)

### Theme 3

#### The importance of social and professional support

The final theme encompasses data that illustrate that social, emotional and informational support from professionals is perceived by parents to be essential to their well-being and ability to cope with stress. This theme consisted of three subthemes: Social Support, Feeling Isolated and Professional Support.

##### Subtheme 1: Social Support

Social support included partners, family members, friends, and in-person and online support groups. Parents often relied on these social supports as coping mechanisms. For example,

The support groups are the only places that you can go where you can talk to people who get it. (Participant 1)

There are two online groups that I’m a part of. . . I have found that parents with children who are now older have wonderful wisdom to actually pass on to me. (Participant 2)

Talking to other people who are walking in the same shoes as you, you get so much from them. So, I find that brilliant. (Participant 4)

It can be so lonely. . . you sometimes feel like. . . you are in a prison. . . but when you have a really bad day. . . just to know that other people are there too. (Participant 5)

Two participants (participants 3 and 6) shared that they often swap with their husbands when there is a situation that becomes extremely challenging so that they may recharge and regain control over their emotional state.

We just say, okay, I need you to take over, and the other person can just walk away and just go take a few deep breaths. . . So we share the balance and, yeah, we adapt. (Participant 3)

My husband and I will swap off. . . I can go up and read a book. . . we try and have our own kind of headspace time because it’s. . . intense’. (Participant 6)

Other parents relied heavily upon friendships to support their own mental health and well-being. Parents surround themselves with friends whom they feel comfortable sharing their difficulties and challenges with, and doing so enables them to feel recharged. For example, some participants stated,

I get away with the girls sometimes overnight or whatever. And I come back better for it. (Participant 4)

If there is a bad day, I suppose I’ve so many friends and support that I’d be vocal. . . sometimes I find the best thing is maybe meeting someone for a coffee and talking about it, and then you feel better. (Participant 5)

##### Subtheme 2: Feeling Isolated (societal stigma)

Some participants said that due to having an autistic child they felt more isolated. They considered this due to preconceived notions regarding the condition. The attitudes of other people, family members and those in wider society made it harder to gain the necessary social support.

Here’s the isolation piece. Family drift away and aren’t that supportive. . . your circle of friends diminishes probably to zero at some point. (Participant 1)

. . . if (name of children) had a broken leg. . . people would instantly be trying to help. . . Autism doesn’t bring that out in other people because people can’t see it. . . all people see is misbehaving. . . we’ve had people say it to us in the supermarket, ‘reign that child in’. . . that’s hard. (Participant 2)

Participants expressed that society needs to have a more positive view of autism as a condition and eradicate negative stereotypes, so that as a nation we can be more inclusive and offer more informal support.

. . . I now feel like I’m on the outside of society. . . I feel that (name of child) is on the outside of society. . . it’s really difficult to be on the outside looking in and feeling shunned. (Participant 1)

I think I’d really love to see a system that is. . . more inclusive. . . making Ireland half for neurodiverse folks and half for neurotypical, make it. . . more inclusive. (Participant 3)

##### Subtheme 3: Professional Support

Despite experiencing stress when trying to access services, many parents were pleased with the quality and deliverance of the support they received from healthcare professionals. Some parents greatly appreciated the professionals’ manner when it came to one-on-one therapies.

I’ve met lovely people. I’ve met lovely individuals. The professionals individually are nice people. . . (Participant 2)

They’re fantastic! (Participant 4)

Parents also found informational courses on how to manage their children’s behaviours extremely helpful. These courses allowed parents to help children achieve important developmental milestones and improve their communication skills.

To be fair to the system, I have learned a huge amount by attending loads of courses to help me to parent my child. (Participant 1)

The first support I had was probably ‘Hanen’, the ‘more than words’ [a program aimed at helping parents improve their autistic child’s communication skills], I found that really good when [name of child] wasn’t verbal. . . I found that brilliant. (Participant 5)

However, parents criticised the informational sessions, believing that the system was teaching them to carry out professional practices (e.g. taking on the role of a speech and language therapist), to substitute receiving guidance from actual professionals.

I see those courses as supplementary to the type of supports that we need. . . they’re training me to do the bits that they’re not doing. . . to be a speech and language therapist. (Participant 1)

They’re not doing one-on-*s someone to hand me a book*. . . one speech and language; they’re teaching the parent how to do it. (Participant 4)

Parents reported that their experiences with schools have been mainly positive and that many teachers and Special Needs Assistants understand the child’s needs and react appropriately.

The supports are wonderful they are great. . . it’s three teachers to six children. . . their sensory profile is known. . . they get their movement breaks, their OT time. . . it’s one on one. (Participant 2)

One participant expressed extreme gratitude in sharing how instrumental the teachers were in helping to toilet-train her child.

His toilet training took a lot longer than other kids. . . [the] teacher was fantastic. . . I wouldn’t have known where to start. (Participant 4)

However, another parent (participant 3) mentioned that while they considered the school to have great resources, there appeared to be a lack of communication between resource teachers and the class teachers causing the participants’ child to become frequently dysregulated. This took ‘hours of decompression’ and increased the child’s anxieties around school, causing distress.

We had a recent incident where my daughter self-harmed. . . it was the miscommunication. . . Her resource teacher had given her a sensory tool to use when she feels dysregulated, and her class teacher took it away from her and she was very dysregulated. . . it was apparent to us that the teacher actually hasn’t read her report. (Participant 3)

## Discussion

### Main findings

This study investigated the challenges that parents of autistic children experience, parental perceptions regarding the service provision and how those challenges and services impact their stress and quality of life. Three key themes were identified: ‘The Autism Journey: Challenges and Rewards’, ‘Navigating a Flawed Support System’ and ‘The Importance of Social and Professional Supports’.

All the participants had similar struggles dealing with difficult behaviours which caused them considerable amounts of distress and significantly impacted their well-being. However, parents were keen to convey that the most ‘challenging’ and ‘stressful’ part of caring for an autistic child was experienced when dealing with autism-based support and services. These findings are consistent with those of Roddy and O’Neill (2020) that found a significant level of economic hardship and unmet need and that 74% of Irish families (n = 195), from data collected in 2014/2015, had not received services in the preceding 12 months. Participants stated that trying to access services was arguably the biggest stressor they encountered on their parenting journey.

The study also found that even though parenting an autistic child can be a challenging experience, it can also be a rewarding and positive one, mirroring other studies (Čolić et al., 2019; Manning et al., 2011; Russell & Norwich, 2012). Positive thinking, recognition of developmental progression and the child’s natural loving disposition were identified as factors that helped parents to recognise that parenting a child with autism is an extremely rewarding experience and one they ‘wouldn’t change for the world’.

### Challenging/difficult situations

Each parent spoke at length about the daily occurrences of their child’s challenging/difficult behaviours, with the biggest problem behaviours being ‘meltdowns’, dangerous behaviours (e.g. self-injurious) and aggression towards others. Many parents described these behaviours as ‘exhausting’ which often left them feeling overwhelmed. These autism-associated behaviours are the most problematic behaviours exhibited by autistic individuals (Hampton et al., 2021; Matheis et al., 2017).

Parents expressed feelings of apprehension surrounding their child’s behaviours as they could not predict how their child would behave in different environments. Unpredictability and uncertainty of dangerous behaviours were major stressors. This finding concurs with those of Ilias et al. (2018) that claims that unpredictable and inappropriate behaviours in autistic children significantly increase parental stress. Constantly elevated stress levels are concerning, with literature suggesting that parents of autistic children are particularly vulnerable to mental health problems due to high levels of depressive symptoms and psychological distress (Picardi et al., 2018).

Negative judgements and a lack of understanding from family and friends were all triggers of parental stress and it caused parents to feel isolated. Indeed, it was found that the level of awareness surrounding autism was poor. Public attitudes are damaging to parents’ mental health and well-being (Alshaigi et al., 2020). Most parents argued that their stress levels would be reduced, and they would feel more included in society through increased acceptance of autism.

### The impact of professional support services on parental stress

The study found that the current state of the HSE in Ireland regarding autism, as experienced by service users, is ‘flawed’ and is not operating in a logistical manner. Parents have described that dealing with the system was more challenging and burdensome than the condition itself. This finding is consistent with Keenan and colleagues’ (2010) study, which described that the system was unclear, distressing and unhelpful. Filling out large amounts of paperwork to access desired services was a stressor that elicited frustration and anger in some participants, as was the lack of follow-through in providing support services.

Due to the high volume of applications for autism-related therapies, and the system’s lack of resources to deliver adequate services, many parents were placed on extensive waiting lists. Parents were subjected to wait unreasonable amounts of time before receiving professional support services (Keenan et al., 2010). It was discovered that the system did not provide any information regarding where the parents’ child was placed on the waiting list. Both the uncertainty of waiting for services to be delivered and the fear of further developmental delays forced many participants to pay for private healthcare services. This caused many parents financial strain, frustration and distress. In other jurisdictions, parents also pay private practitioners to obtain support services, for example, in Taiwan (Chao et al., 2018) and New Zealand (Sainsbury et al., 2023).

According to Malik-Soni et al. (2022), securing an autism diagnosis and diagnosis of comorbidities are the most pressing healthcare needs for an autistic child. This study’s finding that diagnosis can be a delayed, protracted process, that parents find extremely stressful to navigate and to achieve, is very concerning. This situation is also not unique to Ireland (Chao et al., 2018; Sainsbury et al., 2023).

Advocating for their child was key to accessing support services. Participants stressed the importance of frequently following up with services, stating that this ‘disheartening’ process is the only method of accessing support. One notable finding is that the system itself appears to be aware of its inability to provide services of a decent standard, with HSE workers advising caregivers to use their monthly funding from the DCA to access private healthcare. As a result of a negligent system, parents are under huge amounts of avoidable pressure and stress.

Parental stress was also elevated by the lack of information available about what services they should be applying for post-diagnosis. Many of the participants highlighted the need for a guide to be available just after the child has been diagnosed. Essential information was mainly provided by other parents of autistic children through social media platforms. This instilled worry in many parents at the beginning of their journey parenting an autistic child as they had to rely on the experiences of other parents to educate themselves, as opposed to receiving support and guidance from the system itself. According to Ludlow et al. (2012), parents should not have to rely on the Internet to gain valuable information and support.

### Coping strategies

Parents confided in social and professional support to help them cope with challenging situations. Contact with other parents of autistic children was crucial as a form of support (Lai & Oei, 2014; Ludlow et al., 2012). Parents offloaded negative emotions within support groups to parents who understood their struggles. Having a decent support network proved therapeutic for participants and improved parental well-being. Seeking emotional support from partners and friends was also an extremely useful coping mechanism employed by participants.

Informational courses for parents received positive recognition, as they taught parents to deal with challenging behaviours and help their children reach developmental milestones. Many stated that they had learned invaluable techniques to help their child’s development. However, parents highlighted that they felt they were being trained to carry out the jobs of professional practitioners instead of receiving the necessary support from qualified individuals. This finding further illustrates the system’s incapability to deliver adequate support services to autistic children and their parents.

### Recommendations

The amount of service provision in Ireland and its quality are detrimentally affected by the staff recruitment and retention difficulties in the CDNTs. In 2022, the CDNT had a 34% staff vacancy rate (O’Regan, 2023). Therefore, it is imperative that public policy supports the HSE in its efforts to employ and retain CDNT staff and that this work is prioritised.

Parents identified dealing with the system itself was the most challenging part of having an autistic child. Therefore, the aspects of the working processes used by the support systems need to be examined and changed. Future work to determine these changes should employ a public patient partnership approach (www.ppinetwork.ie), to apply international best practice to the Irish context. Work should specifically focus on the following:

1. Reducing the excessive paperwork needed to access services.
2. Reducing parent’s workload chasing services for information about where their child is on waiting lists.
3. Providing structured and timely post-diagnosis information and care pathways that are adequately communicated to parents.

Future work also needs to evaluate and monitor the ongoing effects of system change on parental satisfaction and stress levels and the children’s needs. Some work has been commenced (Cahill et al., n.d.).

## Strengths and limitations of this study

This study addressed a current knowledge gap by providing in-depth data and knowledge about parental experiences. The one-to-one interviews allowed participants to provide comprehensive responses and the comfort to speak openly about their experiences. This openness may not have occurred if interviews were conducted within a group setting.

Although we confirmed data saturation, the small sample size can be regarded as a study limitation. The study is also limited because data were not collected concerning the presence of other conditions that commonly co-occur with autism, including ADHD (Leader et al., 2022), sleep disorders (Whelan et al., 2022), depression and anxiety (Lanyi et al., 2022). These co-occurring conditions would affect the experience of parenting.

## Conclusion

This study revealed that support services in Ireland currently cause parents more distress and challenges than those caused directly by parenting an autistic child. The system is failing to fulfil its main objective, to provide support to autistic individuals and their caregivers. Other less impactful contributors to parental stress were found to be challenging behaviours, societal stigma, isolation, lack of awareness and acceptance of the condition. The findings of this study reveal that both autistic children and their parents are at increased risk of stress due to a flawed system that urgently needs to be better resourced by staffing levels being improved and maintained. System working processes also need to improve to make them more proactive and user-friendly for parents to navigate and so they systematically provide post-diagnosis information.
